# Improved Methodical Approach for Quantitative BRET Analysis of G Protein Coupled Receptor Dimerization

**DOI:** 10.1371/journal.pone.0109503

**Published:** 2014-10-17

**Authors:** Bence Szalai, Péter Hoffmann, Susanne Prokop, László Erdélyi, Péter Várnai, László Hunyady

**Affiliations:** 1 Department of Physiology, Faculty of Medicine, Semmelweis University, Budapest, Hungary; 2 MTA-SE Laboratory of Molecular Physiology, Budapest, Hungary; University of São Paulo, Brazil

## Abstract

G Protein Coupled Receptors (GPCR) can form dimers or higher ordered oligomers, the process of which can remarkably influence the physiological and pharmacological function of these receptors. Quantitative Bioluminescence Resonance Energy Transfer (qBRET) measurements are the gold standards to prove the direct physical interaction between the protomers of presumed GPCR dimers. For the correct interpretation of these experiments, the expression of the energy donor Renilla luciferase labeled receptor has to be maintained constant, which is hard to achieve in expression systems. To analyze the effects of non-constant donor expression on qBRET curves, we performed Monte Carlo simulations. Our results show that the decrease of donor expression can lead to saturation qBRET curves even if the interaction between donor and acceptor labeled receptors is non-specific leading to false interpretation of the dimerization state. We suggest here a new approach to the analysis of qBRET data, when the BRET ratio is plotted as a function of the acceptor labeled receptor expression at various donor receptor expression levels. With this method, we were able to distinguish between dimerization and non-specific interaction when the results of classical qBRET experiments were ambiguous. The simulation results were confirmed experimentally using rapamycin inducible heterodimerization system. We used this new method to investigate the dimerization of various GPCRs, and our data have confirmed the homodimerization of V_2_ vasopressin and CaSR calcium sensing receptors, whereas our data argue against the heterodimerization of these receptors with other studied GPCRs, including type I and II angiotensin, β_2_ adrenergic and CB_1_ cannabinoid receptors.

## Introduction

G Protein Coupled Receptors (GPCRs) were thought to be monomeric entities for a long time, but results of the last two decades indicate that they can form dimers or higher ordered oligomers [1]. Dimerization can alter the ligand binding and active conformation of the receptors, and also the interactions with different effector proteins such as heterotrimeric G proteins and β-arrestins. The effects of dimerization on receptor signaling are proposed to have great physiological and pharmacological consequences [2]–[4]. While the dimerization of Class C GPCRs, including GABAB receptors is widely accepted [5], the occurrence and functional consequences of rhodopsin like Class A GPCR dimerization are more controversial. However, large amounts of data argue that Class A GPCRs can also form dimers, even in native tissues [6], [7], and this dimerization has important effects on receptor function [8], [9].

A wide range of approaches has been used to prove the direct physical interactions between the protomers of a presumed dimer. While some elegant new methods, such as analysis of receptor mobility [10] and visualization of single fluorescently labeled receptors on cell surface [11] are currently available, the current gold standard to study the quaternary structure of GPCRs is the method of quantitative Bioluminescence Resonance Energy Transfer (qBRET) [12], [13].

In qBRET experiments the protomers of the presumed dimer are labeled with the energy donor Renilla luciferase (Rluc) and an energy acceptor fluorescent protein, respectively. Although the energy donor in BRET is the oxidation of coelenterazine h, the substrate of Rluc, for simplicity we will refer to Rluc as the energy donor in this article. The measured energy transfer is highly sensitive to the distance between donor and acceptor, so BRET ratio (calculated by emission at 530 nm/emission at 485 nm) can be excellently used to monitor protein-protein interactions, such as dimerization. BRET is ideal to measure changes of protein-protein interactions (e.g. binding of effector molecules to an activated receptor [14]), however measuring static interactions is more complicated. Labeled plasma membrane proteins can produce measurable BRET signals due to overexpression and random collisions even in the absence of dimerization. To distinguish between specific interaction (dimerization) and non-specific interaction, in qBRET experiments constant amount of donor labeled receptor is coexpressed with increasing amount of acceptor labeled receptor, and the BRET signal is plotted as a function of acceptor/donor expression ratio [12], [13]. In the case of specific interaction a saturation curve is observed, while non-specific interaction results in a linear relationship. While some recent articles suggest, that qBRET curves must be critically interpreted [15]–[17], qBRET experiments are still the most widely used method to study GPCR oligomerization.

In this study, we performed classical qBRET experiments to investigate the dimerization of various GPCRs. We observed saturation qBRET curves between the majority of receptor pairs, but also found, that maintaining a constant expression of donor labeled receptors is hard to achieve in a transient transfection system. We performed Monte Carlo simulations to investigate the effects of non-constant donor expression on qBRET curves. We found, that the changes of donor levels can lead to saturation qBRET curves also in the absence of dimerization. To verify our simulation results, we performed qBRET experiments with varying donor expression levels in a rapamycin inducible heterodimerization system. Based on our simulation and experimental results we suggest a new method to perform and analyze qBRET experiments. With these changes, we were able to investigate the dimerization state of different GPCRs.

## Materials and Methods

### Materials

Molecular biology enzymes were obtained from Fermentas (Vilnius, Lithuania) and Stratagene (La Jolla, CA). The cDNA of the human arginine vasopressin receptor 2 was purchased from S&T cDNA Resource Center (Rolla, MO, USA). Cell culture dishes and plates for BRET measurements were purchased from Greiner Bio-One GmbH (Kremsmunster, Austria). Cell culture media, Lipofectamine 2000 and coelenterazine h were purchased from Invitrogen (Carlsbad, CA). Rapamycin was obtained from Merck (Darmstadt, Germany). HEK293 cells were from American Type Culture Collection (Manassas, VA).

### Plasmid constructs

To create RLuc and Venus tagged human V_2_ vasopressin receptor (AVPR2, Entrez Gene ID: 554), first the receptor sequence was amplified from cDNA clone, purchased from S&T cDNA Resource Center (Rolla, MO, USA). Then the receptor sequence was subcloned using EcoRI and AgeI restriction enzymes into pEYFP-N1 vector containing super Renilla luciferase [18] or monomeric Venus [19], [20] respectively. To create RLuc and Venus tagged human CaSR calcium sensing receptor (CASR, Entrez Gene ID: 846) construct, first the receptor sequence was amplified from pcDNA3.1 plasmid [21], and then was subcloned using HindIII and AgeI restriction enzymes into pEYFP-N1 vector containing super Renilla luciferase [18] or monomeric Venus [19], [20] respectively. For the plasma membrane targeting of PM2-FKBP-RLuc, we used the N-terminal palmitoylation/myristoylation signal of the Lyn protein (MGCIKSKGKDSAGA). To create this construct, the fluorescent protein of PM2-FKBP-mRFP [22] was replaced with super Renilla luciferase [18]. For the plasma membrane targeting of Venus-FRB-CAAX, we used the C-terminal CAAX motif of kRas protein (KMSKDGKKKKKKSKTKCVIM). To create this construct, first the C-terminal ER localization sequence of SacI phosphatase in CFP-FRB-ER(SacI) [22] was replaced with the sequence of kRas CAAX motif from KR-YFP [23]. Then the fluorescent protein was replaced with monomeric Venus [19], [20]. The creation of Venus tagged rat AT_1a_ angiotensin receptor (Agtr1a, Entrez Gene ID: 24180), human β_2_ adrenergic receptor (ADRB2, Entrez Gene ID: 154) and rat CB_1_ cannabinoid receptor (Cnr1, Entrez Gene ID: 25248) were previously described [24]. Venus tagged rat AT_2_ angiotensin receptor (Agtr2, Entrez Gene ID: 24182) was created by exchanging the sequence of eYFP in AT2R-YFP [25] to the sequence of monomeric Venus [19], [20]. For the expression of cytoplasmic Venus fluorescent protein, pEYFP-N1 vector (Clontech, Mountain View, CA, USA), containing the monomeric Venus sequence was used.

### Cell culture and transfection

The experiments were performed on Human Embryonic Kidney 293 (HEK293) cells. Cells were maintained in Dulbecco’s Modified Eagle Medium (DMEM) supplemented with 10% bovine serum, 100 µg/ml streptomycin and 100 IU/l penicillin in 5% CO_2_ at 37°C. Cells were cultured in plastic dishes and were trypsinized prior to transfection. For transient transfection, cells were plated on poly-lysine-pretreated white 96-well plates at 0.75×10^5^ cells/well densities with 0.5 µg/well total plasmid and 0.5 µl/well Lipofectamine 2000.

### Monte Carlo simulations

Simulations were performed on a 2 dimensional membrane lattice containing 100×100 hexagons with periodic boundary conditions. Monomers of donors and acceptors were randomly placed on the empty hexagons of lattice. Total number of molecules was varied from 200 to 2000. The molecules were moved in each simulation step. For each monomer molecule a random neighboring hexagon was selected to move to. If the selected hexagon was occupied with another molecule, move was rejected, and not repeated. For moving dimers, both protomers had to arrive onto a free hexagon, and falling apart of dimers was not allowed during a move step. Generally, dimers were allowed to move to the same direction and/or rotate around each other. Besides moving, monomers could form dimers and dimers could fall apart into monomers in each simulation step. For each monomer molecule one neighbor was randomly selected (if there was any), and the two molecules could form a dimer with a p_association_ probability depending in the type molecules. Dimers could dissociate in each time step with a probability p_dissociation_, also depending on the protomers of the dimer. Simulation was performed for 1000 time steps, and BRET values were calculated. Simulated BRET values were calculated as total neighboring donor-acceptor pairs/number of donor molecules. Each simulation was repeated five times. Simulations were written in Python 2.7. Source code is available online.

### BRET measurements

BRET measurements were performed 24–28 hours after transfection. Medium of the cells was changed to a modified Krebs-Ringer buffer containing 120 mM NaCl, 4.7 mM KCl, 1.2 mM CaCl_2_, 0.7 mM MgSO_4_, 10 mM glucose and 10 mM Sodium-Hepes, pH 7.4. Measurements were performed in a Varioskan Flash Multimode Reader (Thermo Scientific, Waltham, MA). Venus fluorescence (excitation 510 nm, emission 530 nm) was measured at the beginning of each experiment. Coelenterazine h was added at 5 µM final concentration, and total luminescence, luminescence at 485 nm and luminescence at 530 nm were recorded. Intensity ratio was defined as Venus fluorescence/total luminescence. Intensity ratio does not show directly the acceptor/donor ratio, but is proportional with it. From the signals of cells expressing the same amount of RLuc and Venus labeled construct, we approximated that the 1.0 intensity ratio represents an acceptor/donor ratio 1∶1. BRET ratio was calculated as Emission_530_/Emission_485_. The BRET ratio of only donor expressing cells was subtracted from the BRET ratio of measured points. In the inducible dimerization experiments, 100 nM rapamycin or vehicle DMSO was added to the cell medium 30 minutes prior the measurements.

### Figures, statistical analysis

Figures, curve fitting and statistical analysis were performed in GraphPad Prism. All measurements were repeated from n = 3 to 8 (different transfections), and pooled data were plotted. For GPCR dimerization experiments slope of the linear regression for different GPCR pairs was determined for low and high donor expressions, and the difference between slopes was determined by analysis of covariance (ANCOVA). *** indicates significant (p<0.001) difference of slope between the low and high amount donor expressing cells.

## Results

### Analysis of the dimerization of GPCRs using classical qBRET experiments

**Figure 1:**
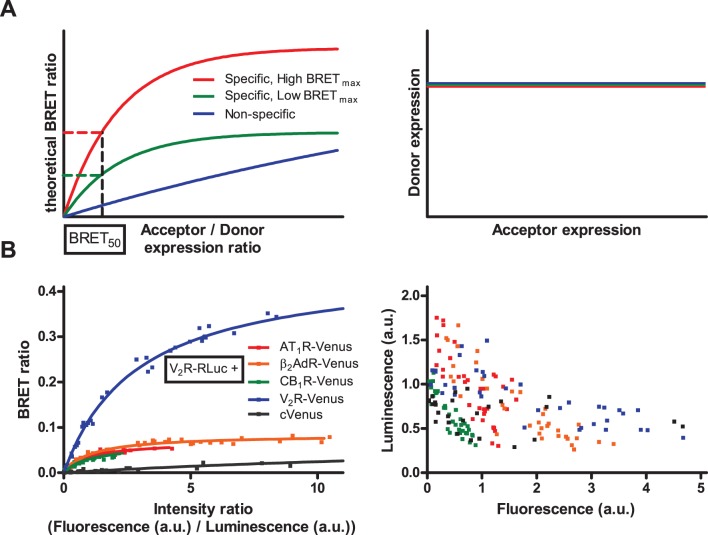
Classical qBRET experiments. (A) Schematic representation of qBRET experiments (based on [12]): In qBRET experiments, constant amount of energy donor labeled receptor is coexpressed with increasing amount of acceptor labeled receptor. BRET ratio is plotted as a function of acceptor/donor expression ratio (left panel). Theoretically specific interactions result in a saturation curve (red and green), while non-specific interaction shows linear relationship (blue). The absolute value of BRET ratio is not indicative of the dimerization state of the receptors, therefore BRET_50_ value (acceptor/donor ratio at half-maximal BRET ratio) is used to determine the affinity of receptors to form dimers (which is the same for red and green curve, indicating the same likelihood of dimerization despite the different BRET_max_ values). To correctly interpret qBRET curves, donor labeled receptor expression has to be maintained constant with increasing acceptor expression (right panel). (B) HEK293 cells were transiently transfected with a constant amount V_2_R-RLuc (donor) coding plasmid and with increasing amounts of either AT_1_R-Venus, β_2_AdR-Venus, CB_1_R-Venus, V_2_R-Venus or cytoplasmic Venus (acceptor) coding plasmid. Various amounts of empty pcDNA3.1 plasmid was added to maintain constant total transfected plasmid amount. Total luminescence and Venus fluorescence were measured at the beginning of each experiment, and intensity ratio was calculated as fluorescence/total luminescence. Intensity ratio shows not the absolute acceptor/donor expression ratio (see Methods for further details) but is proportional with it. BRET ratio was calculated as Emission_530_/Emission_485_, and was plotted as a function of intensity ratio (left panel). Measured total luminescence was plotted as a function of measured fluorescence for the investigated donor - acceptor pairs (right panel). Curves were fitted using non-linear regression equation assuming a single binding site (GraphPad Prism). n = 3.

In classical qBRET experiments, cells are transfected with constant amount of plasmid coding donor labeled receptor, and increasing amount of plasmid coding acceptor labeled receptor [12], [13]. To maintain equal transfection efficacy, total transfected plasmid amount must also be held constant, with the addition of a non-coding plasmid. The measured BRET ratio is plotted as a function of acceptor/donor expression ratio. In this plotting, specific interactions result in a saturation curve, while non-specific interactions lead to a linear relationship (Fig. 1A, left panel). The absolute value of BRET ratio is also dependent on the distance between donor and acceptor in the quaternary complex, so it is not thought to be indicative about the oligomerization state. Therefore the so called BRET_50_ value (acceptor/donor ratio at half-maximal BRET value) is used to determine the presence of dimerization: in experiments conducted with one donor and different acceptor labeled receptors, pairs with low BRET_50_ value thought to form oligomers, while high BRET_50_ values indicate weak interaction or the absence of interaction between the investigated receptors. To correctly interpret qBRET curves, it is necessary to hold constant the donor labeled receptor expression, independent of the expression of acceptor labeled receptors (Fig. 1A, right panel).

We investigated the dimerization of V_2_ vasopressin receptor (V_2_R) with type I angiotensin receptor (AT_1_R), β_2_ adrenergic receptor (β_2_AdR), CB_1_ cannabinoid receptor (CB_1_R) and V_2_R in transiently transfected HEK293 cells. We also used cytoplasmic expressed Venus protein as a negative control. Cells were co-transfected with constant amount of Renilla luciferase labeled V_2_R (V_2_R-RLuc) and increasing amount of the partner receptor labeled with Venus fluorescent protein (AT_1_R-Venus, β_2_AdR-Venus, CB_1_R-Venus and V_2_R-Venus, cVenus respectively). To maintain transfected plasmid amount constant, empty pcDNA3.1 plasmid was added. The calculated BRET ratios were plotted as a function of intensity ratio (measured fluorescence/measured total luminescence), proportional with acceptor labeled receptor expression/donor labeled receptor expression (Fig. 1B, left panel). While the absolute values of BRET ratios were different, the calculated BRET_50_ values for GPCR dimers were in the same order of magnitude (Table 1). The BRET_50_ values indicate the same ability of V_2_R to form dimers with AT_1_R, β_2_AdR and CB_1_R, a lower ability to form homodimers and the absence of specific interaction with cytoplasmic Venus protein. In these experiments, despite the constant amount of transfected V_2_R-RLuc plasmids, we observed a decrease of the measured luminescence with increasing fluorescence levels (Fig. 1B, right panel), indicating that maintaining constant donor labeled receptor expression was not successful. To correctly analyze the results of qBRET experiments, it is necessary to hold donor labeled receptor expression constant, so we could not unambiguously determine the dimerization state of the investigated receptor pairs.

**Table 1:** Calculated BRET_max_ and BRET_50_ values for classical qBRET experiments.

Acceptor	AT_1_R	β_2_AdR	CB_1_R	V_2_R	cVenus
BRET_max_	0.068	0.083	0.059	0.457	0.088
	+/−0.003	+/−0.002	+/−0.005	+/−0.010	+/−0.011
BRET_50_	0.985	1.092	0.962	2.903	26.220
	+/−0.116	+/−0.100	+/−0.192	+/−0.163	+/−6.186

BRET_max_ and BRET_50_ values were calculated for experimental data (Figure 1B, left panel) using a non-linear regression equation assuming a single binding site (GraphPad Prism). Data are given as best fit value +/− Std. Error.

### Monte Carlo simulations to investigate the effects of non-constant donor expression levels on qBRET curves

**Figure 2:**
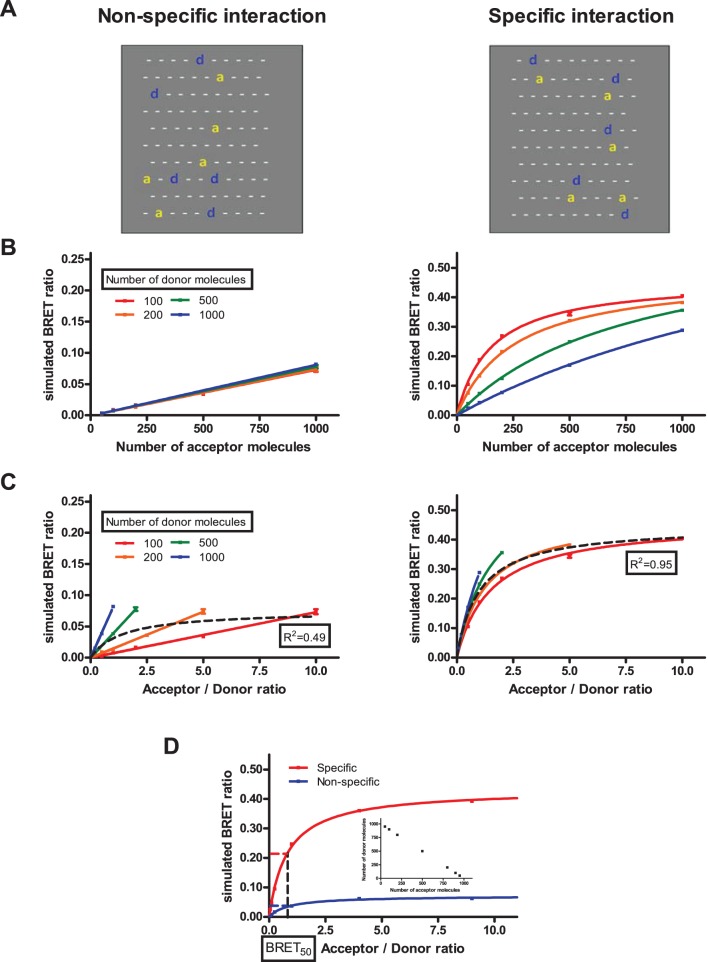
Monte Carlo simulations with various donor amounts. (A) Graphical representation of our simulations: two small membrane pieces in the case of non-specific (left panel) and specific (right panel) interaction, ‘d’ and ‘a’ represents donor and acceptor molecules, respectively, while ‘−‘ indicates empty hexagons. (B and C) Numbers of donor and acceptor molecules were varied from 100 to 1000 for each. BRET ratio was calculated after 1000 simulation time steps based on the total number of neighboring donor-acceptor pairs. Simulated BRET ratio was plotted as a function of the number of acceptor molecules (B) or acceptor/donor ratio (C). Simulations were performed with association and dissociation probabilities for non-specific (left panels) and specific (right panels) interactions (Table 2). (D) Simulations performed for non-specific and specific interactions, when total amount of donor and acceptor were fixed (insert). Curves were fitted using either linear regression or non-linear regression equation assuming a single binding site (GraphPad Prism). n = 5, mean +/− SEM.

To analyze the effect of non-constant donor level on qBRET curves, we performed Monte Carlo simulations. To simulate dimerization, we used the model of Lindermann et al. [26], [27] with little modifications. Briefly, a membrane lattice containing 100×100 hexagons with periodic boundary conditions was created, and monomers of donor and acceptor molecules were randomly placed. The molecules could move and dimerize in each time step of the simulation. If a monomer molecule had a neighbor molecule, they could dimerize with a probability (p_association_) dependent of the types of the molecules. Dimers had a probability (p_dissociation_) to fall apart in each simulation step, which is dependent on the type of the dimer. After 1000 time steps, simulated BRET ratios were calculated based on the number of neighboring donor – acceptor pairs (donor acceptor pairs could be formed by dimerization or by randomly moving a donor and an acceptor molecule to neighboring hexagons). To specify the type of interaction between donor and acceptor molecules we used different p_association_ and p_dissociation_ values (Table 2). A graphical representation of our simulation experiments with a small membrane piece is shown in Figure 2A. Further details of the Monte Carlo simulations are found in Methods. In our simulations, we varied both the number of donor and acceptor molecules from 100 to 1000. We used two different representations of the data: the simulated BRET ratio was plotted as function of the number of acceptor molecules (Fig. 2B, Type I plots) or as function of the acceptor/donor ratio (Fig. 2C, Type II plots, same as classical qBRET plots). In the case of non-specific interaction we found that the simulated BRET ratio is only dependent on the amount of acceptor molecules (Fig. 2B, left panel), whereas the amount of donor molecules has no influence on the BRET value. This phenomenon is reflected in Type II plots by the linear relationship between acceptor/donor ratio and BRET ratio, but in this case the slope of the linear regression is dependent on the number of donor molecules (Fig. 2C, left panel). In the case of specific interaction, the BRET ratio is dependent both on the amount of acceptor and donor molecules. Type I plot (Fig. 2B, right panel) shows that increasing the number of acceptors leads to an increased BRET ratio, and this increase is reduced, when the number of donor molecules is high. In Type II plot (Fig. 2C, right panel) the BRET ratios show different saturation curves, which are dependent on the donor amount. When data points were not separated by donor expression in Type II plots, we could fit a reasonable one-site specific binding curve for specific interaction (R^2^ = 0.95), while this was not possible for non-specific interaction (R^2^ = 0.49) (black dashed curve).

**Table 2:** Simulation parameters: Association and dissociation probabilities used for the simulation of non-specific and specific interactions.

Type of interaction	Non-specificinteraction	Specificinteraction
Donor – Donor	p_association_ = 0.0	p_association_ = 0.0
	p_dissociation_ = 0.0	p_dissociation_ = 0.0
Donor – Acceptor	p_association_ = 0.0	p_association_ = 1.0
	p_dissociation_ = 0.0	p_dissociation_ = 0.0
Acceptor – Acceptor	p_association_ = 0.0	p_association_ = 0.0
	p_dissociation_ = 0.0	p_dissociation_ = 0.0

To model the reducing effect of increased acceptor expression on donor expression (Fig. 1B, right panel), which caused problems for the analysis of our initial classical qBRET experiments, we performed simulations where the total amounts of donor and acceptor molecules were held constant (Fig. 2D, insert). In this case, specific and non-specific interactions both led to saturation curves with nearly identical BRET_50_ values in Type II plots (Fig. 2D). These data show that when donor expression decreases with increasing acceptor expression, the dimerization state cannot be determined using the classical presentation of qBRET curves.

### The effects of non-constant donor expression levels on qBRET curves in an inducible dimerization system

**Figure 3:**
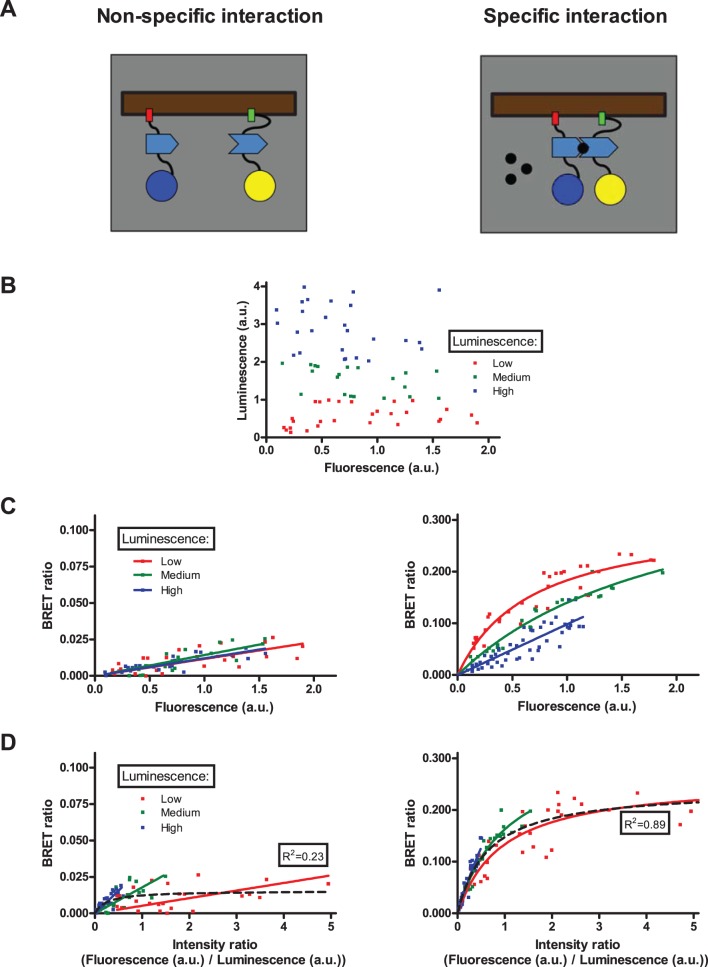
Rapamycin inducible dimerization with various donor amounts. (A) Schematic representation of the rapamycin inducible dimerization system: PM2-FKBP-RLuc (left molecule) and Venus-FRB-CAAX (right molecule) show non-specific interaction in the absence of rapamycin (left panel), while the presence of rapamycin (right panel) results the dimerization of FKBP and FRB domains, converting the interaction into specific. (B, C and D) HEK293 cells were transiently transfected with increasing amounts of PM2-FKBP-RLuc and Venus-FRB-CAAX, while empty pcDNA3.1 plasmid was added to maintain total transfected plasmid amount constant. Fluorescence-Luminescence plot (B) shows a wide and independent distribution of acceptor and donor expression. Cells were treated with vehicle (left panels) or 100 nM rapamycin (right panels) 30 minutes prior to measurements. Total luminescence and Venus fluorescence were measured at the beginning of each experiment, and intensity ratio was calculated as fluorescence/total luminescence. BRET ratio was calculated as Emission_530_/Emission_485_, and was plotted as a function of measured fluorescence (C) or intensity ratio (D). Measured points were sorted into low/medium/high luminescence groups based on the measured total luminescence (B). Curves were fitted using either linear regression or non-linear regression equation assuming a single binding site (GraphPad Prism). n = 3.

To experimentally verify our simulation results, we have taken advantage of the well characterized rapamycin-induced dimerization of FKBP and FRB protein domains [28]. Two non-interacting plasma membrane markers, PM2 and CAAX were labeled with FKBP/FRB and RLuc/Venus, respectively (PM2-FKBP-RLuc and Venus-FRB-CAAX). We choose these markers, because they are expressed in the plasma membrane, similar to GPCRs. PM2 and CAAX has no basal interaction, since they are localized in different plasma membrane microdomains [20]. In the absence of rapamycin only non-specific interaction can be observed, since the donor and acceptor do not interact (Fig. 3A, left panel). Addition of rapamycin (100 nM, 30 min) leads to dimerization of FKBP and FRB, which causes specific interaction between the donor and acceptor molecules (Fig. 3A, right panel).

HEK293 cells were co-transfected with increasing amount of PM2-FKBP-RLuc and Venus-FRB-CAAX coding plasmids, and empty pcDNA3.1 plasmid to reach constant total transfected plasmid amount. The amount of plasmids encoding donor and acceptor proteins was independently varied (from 0.002 µg/well to 0.02 µg/well). In this way, we could reach a wide range of different donor and acceptor expression levels (Fig. 3B). Measured points were sorted into low, medium and high luminescence groups based on the measured total luminescence (representing donor expression). Similar to the presentation of our simulation results, we used two representations of our experimental data. BRET ratio was plotted either as a function of measured fluorescence, representing acceptor expression (Fig. 3C, Type I plots) or as a function of measured fluorescence/measured total luminescence (Fig. 3D, Type II plots).

The experimental results were in good agreement with the modeling data generated by Monte Carlo simulations. Type I plot shows that in the case of non-specific interaction, the BRET ratio is only dependent on the expression of acceptor labeled construct (Fig. 3C, left panel). However, we found that in the case of specific interaction, increasing the donor expression leads to flattening of the qBRET curve (Fig. 3C, right panel). In Type II plots, we found different linear and saturation curves for non-specific and specific interaction, respectively (Fig. 3D). Similar to our simulations, when the data points were not separated by donor expression, we could fit a saturation curve on the specific interaction data (R^2^ = 0.89), but could not make this for non-specific interaction (R^2^ = 0.23) in the Type II plots (black dashed curve).

### Modified qBRET experiments to analyze the dimerization of GPCRs

Based on our simulation and experimental results, we analyzed the dimerization of different GPCRs by a modified version of classical qBRET experiments. Instead of trying to maintain constant expression of the donor labeled receptor, we varied the transfected amount of donor and acceptor labeled receptor as well, to reach a wide range of different donor – acceptor expressions. Based on our results, to distinguish specific and non-specific interaction, it is more appropriate to plot BRET ratio as a function of measured fluorescence (acceptor expression) (see Figure S1. for further details). While classical qBRET experiments require high level of acceptor expression to reach saturation curves, our method allowed us to differentiate between specific and non-specific interaction also at lower acceptor expression levels, on the initial lineal phase of the curves. In the case of non-specific interaction, BRET ratio is only dependent on the acceptor expression, and independent of the donor expression, the measured BRET ratios are on the same flat linear curve (Fig. 2B and 3C, left panels). In the case of specific interaction the fluorescence-BRET ratio plot is steeper, and the increase of donor expression leads to a flattening of the curve (Fig. 2B and 3C, right panels).

**Figure 4:**
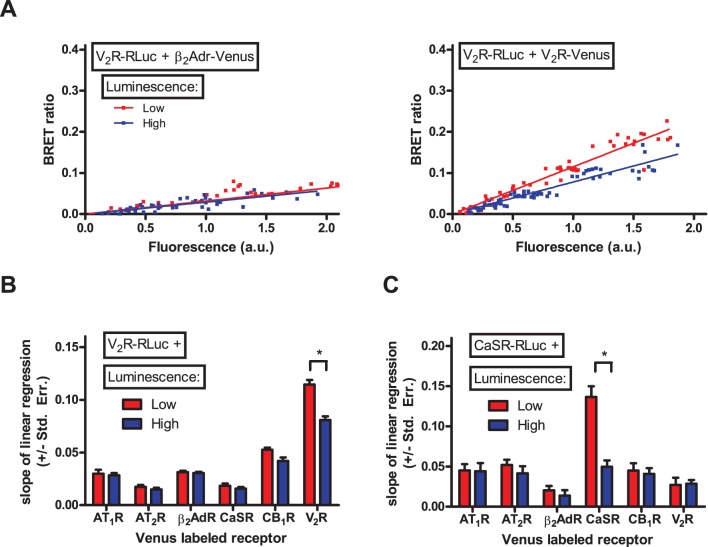
Dimerization of V_2_ vasopressin receptor and CaSR calcium sensing receptor with various other GPCRs. HEK293 cells were transiently transfected with increasing amounts of V_2_R-RLuc (A and B) or CaSR-RLuc (C) and with increasing amounts of either AT_1_R-Venus, AT_2_R-Venus, β_2_AdR-Venus, CaSR-Venus, CB_1_R-Venus or V_2_R-Venus. Various amounts of empty pcDNA3.1 plasmid was added to maintain constant total transfected plasmid amount. Total luminescence and Venus fluorescence were measured at the beginning of each experiment. BRET ratio was calculated as Emission_530_/Emission_485_, and was plotted as a function of measured fluorescence. (A) Representative Type I plots for V_2_R-β_2_AdR interaction (left plot) and V_2_R-V_2_R interaction (right plot). Measured points were sorted into low/high luminescence groups based on the total measured luminescence (Figure S2). (B and C) The slope of linear regression was calculated for the low and high luminescence groups of different GPCR pairs, and was plotted as a column diagram. Difference between the slopes of linear regression was determined by ANCOVA. n = 3–8.

We analyzed the dimerization of V_2_R and CaSR calcium sensing receptor (a typical Class A and Class C GPCR, respectively) with AT_1_R, type II angiotensin receptor (AT_2_R), β_2_AdR, CaSR, CB_1_R and V_2_R. HEK293 cells were transfected with increasing amount of donor (V_2_R-RLuc or CaSR-RLuc) and acceptor (AT_1_R-Venus, AT_2_R-Venus, β_2_AdR-Venus, CaSR-Venus, CB_1_R-Venus or V_2_R-Venus) labeled receptor coding plasmid. Empty pcDNA3.1 plasmid was added to reach constant total transfected DNA amount. Measured points were sorted into low and high luminescence groups based on the measured total luminescence, similarly to our inducible dimerization experiments (Figure S2). BRET ratio was plotted as a function of measured fluorescence. Figure 4A shows our results for the V_2_R- β_2_AdR (left panel) and V_2_R-V_2_R (right panel) interaction. In the case of V_2_R- β_2_AdR interaction, the Type I plot shows flat linear relationship; where the donor expression does not influence the slope of the linear regression, indicating the non-specific nature of this interaction. In contrast, in the case of V_2_R-V_2_R interaction, the curve is steeper, and the increase of donor expression leads to flattening of the curve, showing the homodimerization of V_2_R. We performed the same analysis for all of the investigated receptor pairs (Figure S2), and plotted the slope of the linear regression also for low and high amount of donor expression (Fig. 4B and 4C). We found, that the slope of linear regression was low and the donor expression did not influence the slope for all of the investigated receptor pairs, except the V_2_R-V_2_R and the CaSR-CaSR interaction. Our data indicate the homodimerization of V_2_R and the homodimerization of CaSR, and the absence of heterodimerization between the investigated receptors.

## Discussion

Dimerization or oligomerization of receptors is an emerging question of the GPCR field. Dimerization of GPCRs can alter the binding and signaling of GPCRs, and is thus proposed to have major physiological and pharmacological consequences. While many functional data support the concept of dimerization, evidence of direct physical interaction is crucial to declare the dimerization of two GPCRs. The most widely used method for the latter is the method of quantitative BRET.

In qBRET experiments constant amount of donor labeled receptor is expressed, while the amount of acceptor labeled receptor is increased [12], [13]. When BRET ratio is plotted as a function of acceptor/donor amount (Fig. 1A, left panel), specific interaction (dimerization) leads to saturation curves, while non-specific interaction results in a linear relationship (or saturation curve with a high BRET_50_ value). The analysis of qBRET curves supposes that with the increasing acceptor expression, the donor expression does not change (Fig. 1A, right panel). In our classical qBRET experiments, we found that all of the investigated pairs resulted in saturation qBRET curves with BRET_50_ values of the same magnitude, except for cytoplasmic Venus (Fig. 1B, left panel). These data would indicate approximately the same ability of V_2_R to form homodimer and heterodimers with the investigated receptors, and the absence of specific interaction between V_2_R and cytoplasmic Venus. While cytoplasmic fluorescent proteins were originally used [13] as negative controls for qBRET experiments, they are absolutely not ideal controls: only a small fraction of cytoplasmic proteins are in close proximity of the plasma membrane to produce energy transfer signal, so the “effective” acceptor amount is much smaller than the total acceptor amount estimated by measuring fluorescence. This explains the phenomenon that cytoplasmic fluorescent proteins are always resulting in flat qBRET curves. Despite this fact, cytoplasmic proteins are still frequently used as negative controls also nowadays [29].

We also found, that despite the constant amount of donor labeled receptor coding plasmid in the transfection reaction, the increase in acceptor expression leads to the decrease in donor expression (Fig. 1B, right panel). In most of the related articles, the constant expression of donor labeled receptors was only assumed based on the constant amount of the donor labeled receptor coding plasmid in the transfection reaction; however, in most cases the actual expression levels were not shown explicitly. Also the original article describing qBRET method states that “Although the BRET saturation curves were carried out using a fixed concentration of the RLuc fusion partners, co-transfecting an increasing quantity of the GFP constructs introduces some levels of variability in the amount of receptor-RLuc expressed in each case. To rule out the influence of this variable, the BRET levels were plotted as a function of the ratio between the receptor-GFP/receptor-Rluc numbers” [13]. So it can be supposed, that the decrease of donor expression was not only a problem in our experiments, but a general problem of qBRET experiments.

We performed Monte Carlo simulations to investigate the effects of these changes of donor expression on qBRET curves. Simulations were performed in a membrane lattice containing 100×100 hexagons, where one receptor could occupy one hexagon. The total numbers of receptors were varied between 200 and 2000 in the simulations. Assuming that the size of an average cell is 50µm and the diameter of a GPCR is 5 nm, our simulations represent approximately 10^6^ receptors/cell, which are normal values for an overexpression system. We found, that the decrease of donor expression can lead to a saturation qBRET curve also in the case of non-specific interaction (Fig. 2D), which fact can lead to a false interpretation of dimerization state of the investigated receptors. While the strictly inverse relationship between donor and acceptor expression in this simulation seems to be a bit of rigid condition, our classical qBRET experiments showed similar expression alterations (especially for AT_1_R and CB_1_R, Figure 1B, right panel).

Our simulations showed that the difference between non-specific and specific interaction is less ambiguous when the donor and acceptor amount is also altered, and the BRET ratio is plotted as a function of acceptor amount (Fig. 2B). In this presentation, non-specific interaction leads to a flat linear curve, and the amount of donor molecules does not influence the BRET ratio (Fig. 2B, left panel). At the first impression this phenomenon was surprising, but it is easy to interpret. The measured BRET ratio is the average of the BRET ratios of the discrete donor molecules. The BRET ratio of one donor molecule can be interpreted as the average time spent by this donor molecule in the molecular proximity of the acceptor molecules. Increasing the number of acceptor molecules increases this time, so also the BRET ratio. When the number of donor molecules increases, the average time spent by one donor molecule in the molecular proximity of acceptors does not change, so the BRET ratio is not dependent on the number of donor molecules. In the case of specific interaction, the curve is steeper, and the increase in donor amount leads to the flattening of the curve (Fig. 2B, right panel). When the number of donor molecules increases, more donor molecules do not form dimers with acceptors, so the flattening of the curve is easy to interpret.

Alternatively, specific and non-specific interaction is also distinguishable, when the data points are not separated by donor amount, and a one-site specific binding curve is fitted on the acceptor/donor ratio – BRET plots (Fig. 2C, black dashed line). In this case, a reasonable curve fitting is possible for specific interaction (R^2^>0.9), but not for non-specific interaction (R^2^<0.5). However, for the reasons discussed above, this analysis needs a larger amount of data points with ideal acceptor-donor distribution (Figure S1), hence the reason for us not favoring its use in the latter GPCR dimerization experiments.

To experimentally validate the results of our simulation we studied the rapamycin inducible dimerization of plasma membrane targeted FKBP and FRB protein domains. Although many controls have been used previously for qBRET experiments, including known interacting and non-interacting proteins, to our knowledge our results are the first attempt to demonstrate the relevance of qBRET curves in an inducible dimerization system. The clear advantage of this system is that the investigated proteins and their quantities are identical during the non-specific and specific interaction. It is also important, that these proteins are both plasma membrane expressed, thus they are better controls for GPCR dimerization than cytoplasmic proteins that are also used in earlier studies. Our experimental results showed nice correlation with the simulations (Fig. 3C and 3D).

Based on our findings we propose a new methodical approach to qBRET experiments, where various amounts of donor and acceptor constructs are expressed, and the BRET ratio is plotted as a function of the acceptor expression. With these changes, we could clearly distinguish specific and non-specific interactions (Fig. 2B and 3C). In our experiments, we confirmed the previously described homodimerization of V_2_ vasopressin [30]–[33] (Fig. 4B) and CaSR calcium sensing receptors [34]–[38] (Fig. 4C), but found no heterodimerization of these receptors with the GPCRs investigated in our study. These data indicate that the dimerization of GPCRs is a rather specific phenomenon, as we found only two receptor pairs (the two homodimers) to form dimers out of the total twelve investigated receptor pairs. It is important to state that using the classical interpretation of qBRET experiments, our data suggested that V_2_R has similar affinity to form dimers with AT_1_R, β_2_AdR, CB_1_R and V_2_R (Fig. 1B, left panel and Table 1), whereas analysis of these interactions based on our new methodology clearly showed that only the V_2_R-V_2_R interaction is specific (Fig. 4B).

**Figure 5:**
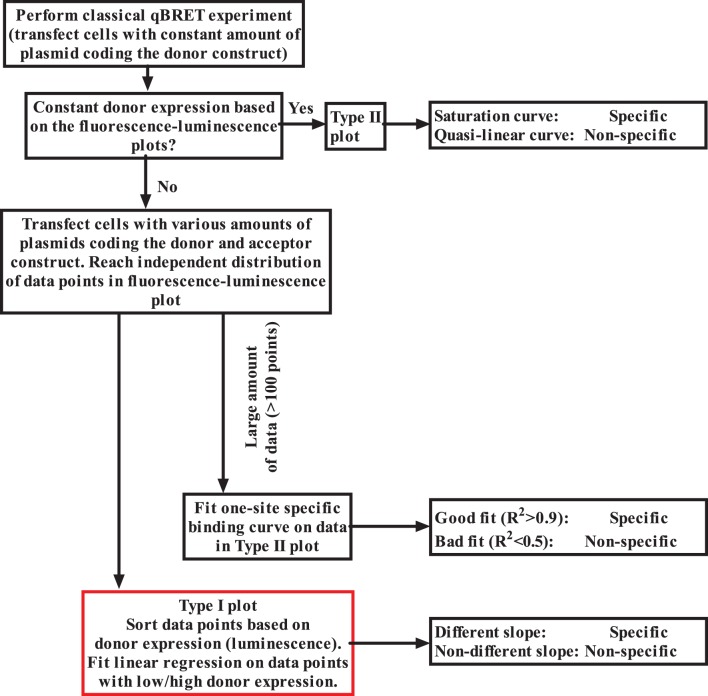
Preferred method to perform qBRET experiments. In Type I plots BRET ratio is plotted as a function of acceptor expression (fluorescence), while in Type II plots BRET ratio is plotted as a function acceptor/donor expression (fluorescence/luminescence).

In this article, we showed that classical qBRET experiments can lead to false results, when the expression of donor labeled receptor is not held strictly constant. Of course, our findings do not mean that all results obtained in previous qBRET experiments are incorrect, since when donor expression falls with increasing acceptor expression levels specific interaction also leads to saturation qBRET curves (Fig. 2D). However, in many cases non-specific interactions can lead to saturation qBRET curves, because donor expression can decrease, when the acceptor expression is increased. Since this problem can lead to false positive interpretation of GPCR dimerization interactions, we suggest that interpretation of earlier qBRET experiments should be reconsidered (Fig. 5). In conclusion, our data suggest that expression of the donor construct must be closely monitored during qBRET analysis of GPCR dimers, and our method provides a solution to exclude misinterpretation of the data caused by alterations of donor receptor expression.

## Supporting Information

Figure S1 Type I plots outperform Type II plots in detecting non-specific interaction: Based on our simulations and inducible dimerization experiments, we propose two different analysis methods for qBRET experiments. Both methods require a wide range of different levels of donor and acceptor expressions (left panels). In the first method (Type I plots, middle panel) BRET ratio is plotted as a function of acceptor expression, and the difference between the slope of linear regression for points with low and high donor amount is investigated. In the second method (Type II plots, right panel) BRET ratio is plotted as a function of acceptor/donor ratio, and one-site specific binding curve is fitted on the whole data. Good (R^2^>0.8) fit suggests specific interaction, while non-specific interaction results in a worse (R^2^<0.5) fit. To investigate the performance of these two methods, we performed additional Monte Carlo simulations. Simulations were ran with random acceptor and donor amount (in the [100∶1000] range) and with simulation parameters for non-specific interaction. After the simulation a random Gaussian noise term was added to the acceptor, donor and BRET values to further approximate our experimental setup. Simulations were performed for n = 100 (A) or n = 20 (B) data points. When data sample is sufficiently large (A) both methods showed the non-specific nature of interaction. However, with smaller sample size, but still with a wide range of different donor-acceptor amounts (B, left panel), it is possible to get such a distribution of data points, where in Type II plots (B, right panel) a reasonable saturation curve can be fitted (suggesting specific interaction). In this case Type I plot still shows correctly the non-specific nature of this interaction. Based on these data, we think that when the data sample is not very large (<100 points), Type I plots can better differentiate between specific and non-specific interaction.
(PDF)

Figure S2 Fluorescence-Luminescence, Type I and Type II plots of GPCR dimerization experiments: HEK293 cells were transfected with various amounts of different donor and acceptor coding plasmids. Measured points were sorted into low/high luminescence groups based on the total measured luminescence (red: low luminescence, blue: high luminescence). Fluorescence-Luminescence (left), Fluorescence-BRET ratio (middle) and Intensity ratio-BRET ratio (right) plots were created for different donor-acceptor pairs. Summary of this plot can be found in Figure 4B and 4C.
(PDF)
