# Correction: Improved Methodical Approach for Quantitative BRET Analysis of G Protein Coupled Receptor Dimerization

**DOI:** 10.1371/journal.pone.0156824

**Published:** 2016-05-31

**Authors:** Bence Szalai, Péter Hoffmann, Susanne Prokop, László Erdélyi, Péter Várnai, László Hunyady

Some technical errors occurred during preparation of the plots for S2 Fig. The plots for the main figures of the paper were prepared using a value of 1.2 a.u. as the threshold to separate the high (blue) and low (red) luminescence points. Due to an error, most of the plots in S2 Fig were created using an incorrect value of 1.25 a.u. as the high/low threshold. The Fluorescence/BRET ratio plot of the V_2_R-RLuc + β_2_AdR-Venus plot in S2 Fig was created using an incorrect value of 1.0 a.u. as the high/low threshold.

Additionally, the underlying raw data set deposited to GitHub had a cut off of 2.0 a.u., which is a limited set of data for the V_2_R-RLuc/V_2_R-Venus and V_2_R-RLuc/β_2_AdR-Venus interactions. The original plots were generated with a maximum Fluorescence axis cut off of 2.1 a.u., which is also a limited set of data for the V_2_R-RLuc/V_2_R-Venus and V_2_R-RLuc/β_2_AdR-Venus interactions. The authors confirm that when the full set of data is used, changes in the luminescence threshold do not affect the conclusion that the interactions between the V_2_R-RLuc and β_2_AdR-Venus are not significant, while the evidence indicates homodimerization of V_2_ receptors.

The authors apologise for the errors, and here we provide a revised Fig 4 created using the full data set, with a Fluorescence axis of 0–3.0 a.u.. We also provide a revised S2 Fig in which plots were created using the full data set, with Fluorescence axes extended to 3.0 a.u. where necessary and using a high/low luminescence threshold of 1.2 a.u.. In all revised plots, the regression line is forced through the origin.

**Fig 4:**
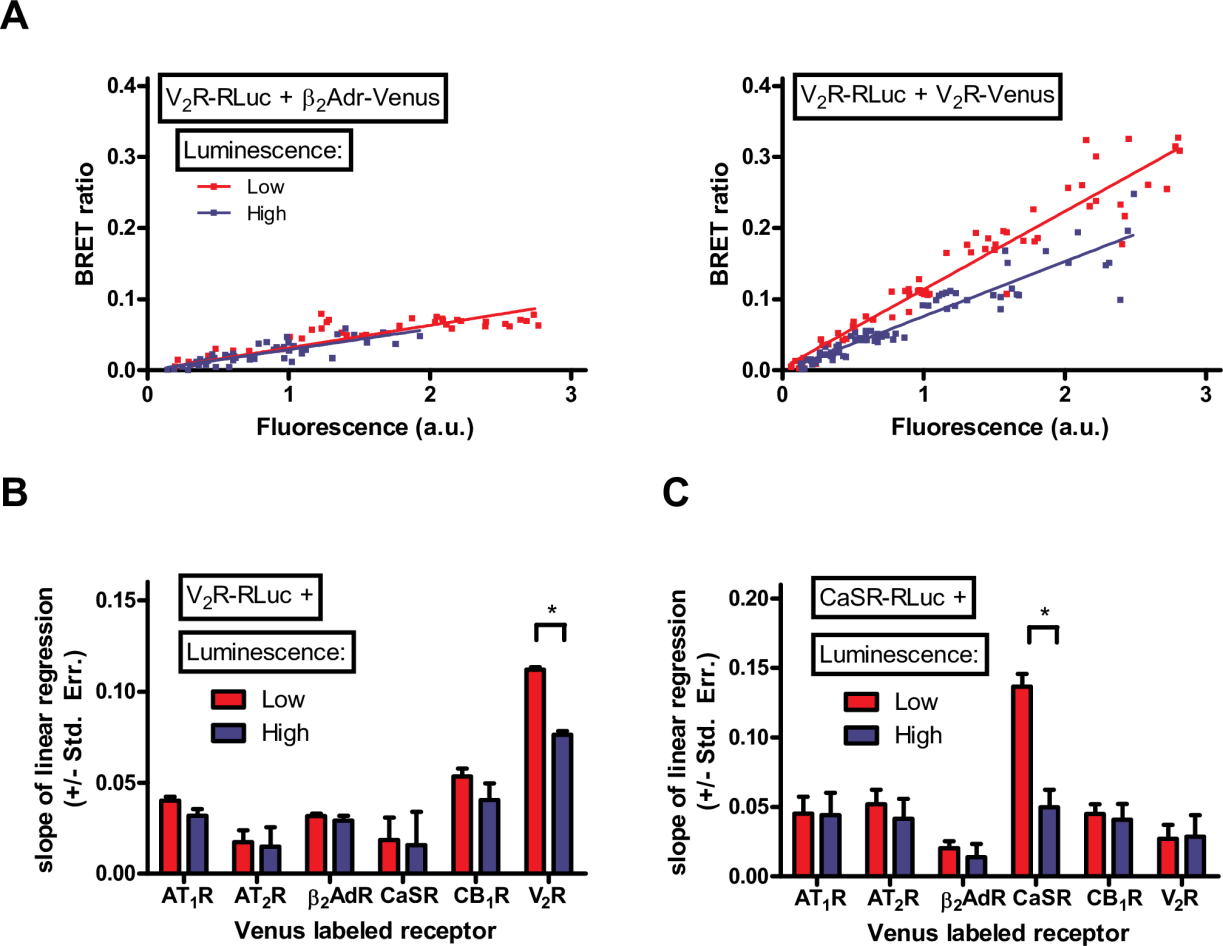
Dimerization of V_2_ vasopressin receptor and CaSR calcium sensing receptor with various other GPCRs. HEK293 cells were transiently transfected with increasing amounts of V_2_R-RLuc (A and B) or CaSR-RLuc (C) and with increasing amounts of either AT_1_R-Venus, AT_2_R-Venus, β_2_AdR-Venus, CaSR-Venus, CB_1_R-Venus or V_2_R-Venus. Various amounts of empty pcDNA3.1 plasmid was added to maintain constant total transfected plasmid amount. Total luminescence and Venus fluorescence were measured at the beginning of each experiment. BRET ratio was calculated as Emission_530_/Emission_485_, and was plotted as a function of measured fluorescence. (A) Representative Type I plots for V_2_R-β_2_AdR interaction (left plot) and V_2_R-V_2_R interaction (right plot). Measured points were sorted into low/high luminescence groups based on the total measured luminescence (S2 Fig). (B and C) The slope of linear regression was calculated for the low and high luminescence groups of different GPCR pairs, and was plotted as a column diagram. Difference between the slopes of linear regression was determined by ANCOVA. n  =  3–8.

Performing the statistical analysis by forcing the regression line through the origin resulted in the p values shown in S1 Table.

New ANOVA analyses were also performed. The “slope” for each data point was obtained by calculating BRET ratio / Fluorescence. The average slope for high and low luminescence groups was calculated, and a two-way ANOVA was performed using these data. Our results are shown in S3 Fig. This alternative analysis also confirmed our previous results (homodimerization of V_2_R and CaSR, and absence of dimerization between the investigated heterodimers).

The full data set and the R script for statistical analysis have been deposited to GitHub as file GPCR2.dat and statistics.R (https://github.com/bence-szalai/Improved-methodical-approach-for-quantitative-BRET-analysis-of-G-protein-coupled-receptor-dimerizati).

## Supporting Information

S1 Table Calculated p values for the difference between the slopes for high/low luminescence points. (DOCX)

S2 Fig Fluorescence-Luminescence, Type I and Type II plots of GPCR dimerization experiments: HEK293 cells were transfected with various amounts of different donor and acceptor coding plasmids. Measured points were sorted into low/high luminescence groups based on the total measured luminescence (red: low luminescence, blue: high luminescence). Fluorescence-Luminescence (left), Fluorescence-BRET ratio (middle) and Intensity ratio-BRET ratio (right) plots were created for different donor-acceptor pairs. Summary of this plot can be found in Fig 4B and 4C.
(PDF)

S3 Fig Analysis of the dimerization of V_2_ vasopressin and CaSR calcium sensing receptor with various other GPCRs using ANOVA. For each data point BRET ratio / Fluorescence ratio was calculated. Two-way ANOVA was performed (acceptor receptor and high/low luminescence as the two factors) with Bonferroni post hoc test on these data to evaluate the effect of high/low luminescence of BRET ratio / Fluorescence. Data are plotted as mean +/- S.E.M. ***: p<0.001
(PDF)
