# Supplementary figures and images for: Improved Methodical Approach for Quantitative BRET Analysis of G Protein Coupled Receptor Dimerization
Source: PLoS One. 2014 Oct 17;9(10):e109503. doi: 10.1371/journal.pone.0109503 (PMC4201472; doi:10.1371/journal.pone.0109503)

Figure S1:

A

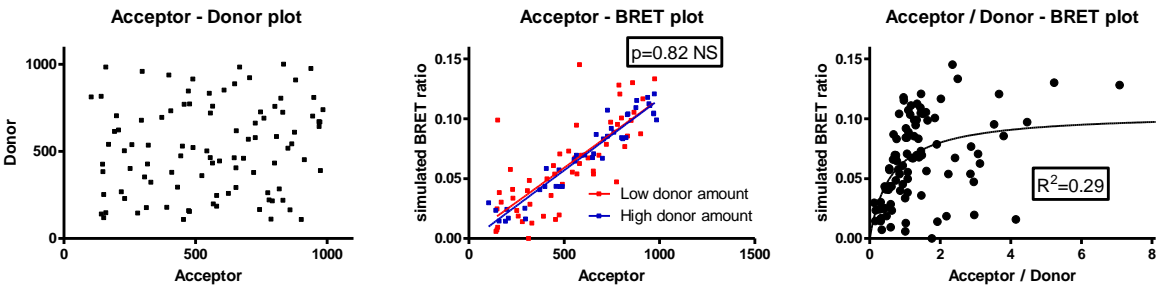

B

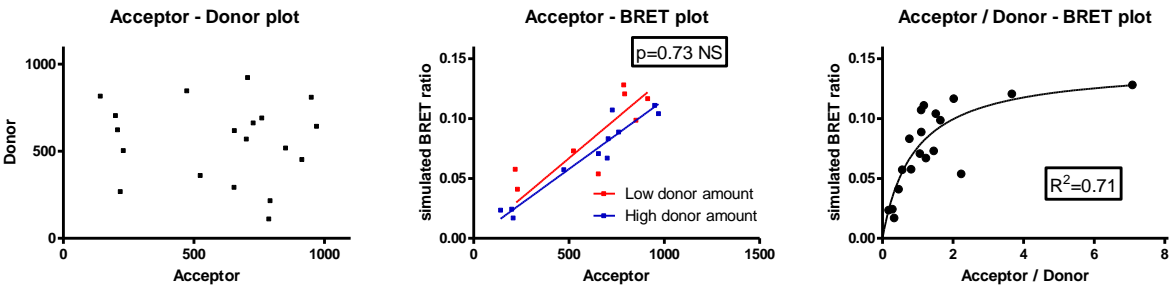

Supplement: Figure S1 — Type I plots outperform Type II plots in detecting non-specific interaction: Based on our simulations and inducible dimerization experiments, we propose two different analysis methods for qBRET experiments. Both methods require a wide range of different levels of donor and acceptor expressions (left panels). In the first method (Type I plots, middle panel) BRET ratio is plotted as a function of acceptor expression, and the difference between the slope of linear regression for points with low and high donor amount is investigated. In the second method (Type II plots, right panel) BRET ratio is plotted as a function of acceptor/donor ratio, and one-site specific binding curve is fitted on the whole data. Good (R2>0.8) fit suggests specific interaction, while non-specific interaction results in a worse (R2<0.5) fit. To investigate the performance of these two methods, we performed additional Monte Carlo simulations. Simulations were ran with random acceptor and donor amount (in the [100∶1000] range) and with simulation parameters for non-specific interaction. After the simulation a random Gaussian noise term was added to the acceptor, donor and BRET values to further approximate our experimental setup. Simulations were performed for n = 100 (A) or n = 20 (B) data points. When data sample is sufficiently large (A) both methods showed the non-specific nature of interaction. However, with smaller sample size, but still with a wide range of different donor-acceptor amounts (B, left panel), it is possible to get such a distribution of data points, where in Type II plots (B, right panel) a reasonable saturation curve can be fitted (suggesting specific interaction). In this case Type I plot still shows correctly the non-specific nature of this interaction. Based on these data, we think that when the data sample is not very large (<100 points), Type I plots can better differentiate between specific and non-specific interaction. (PDF) [file pone.0109503.s001.pdf]

Figure S2:

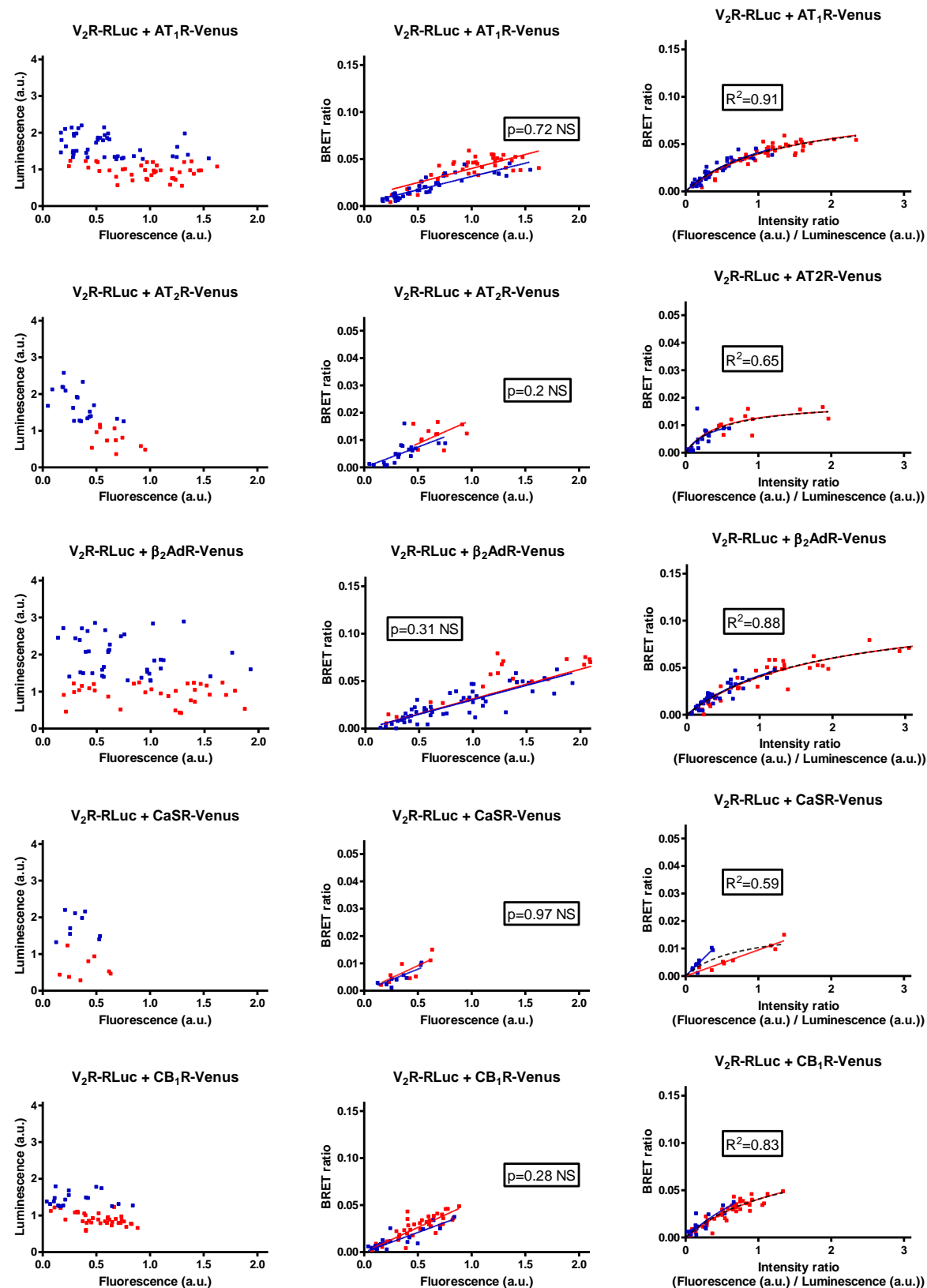

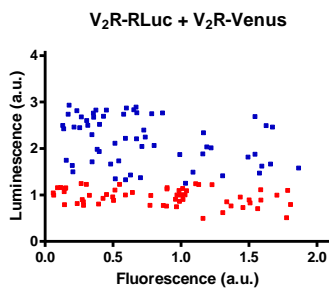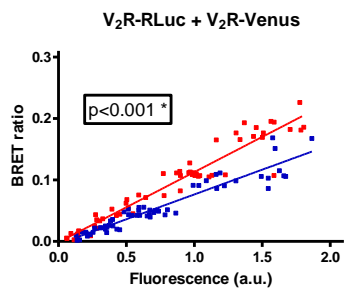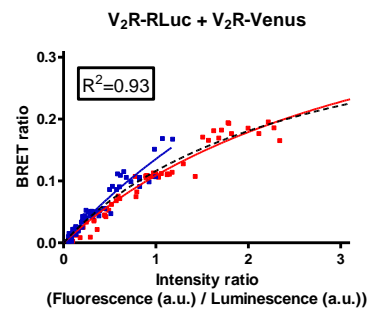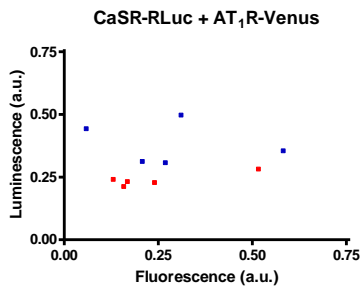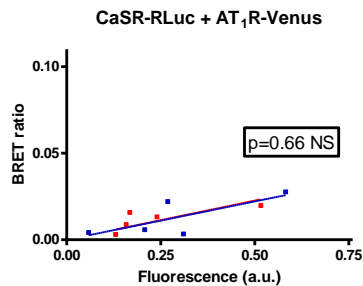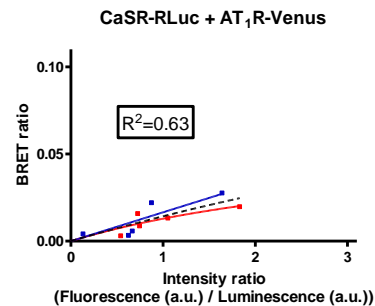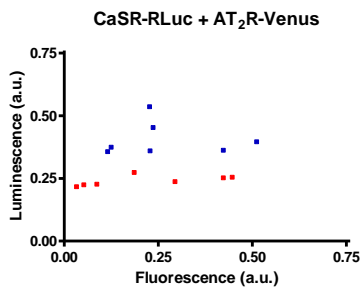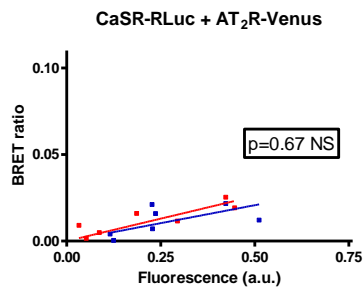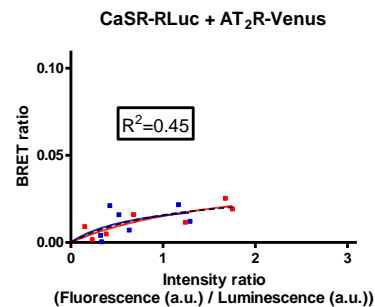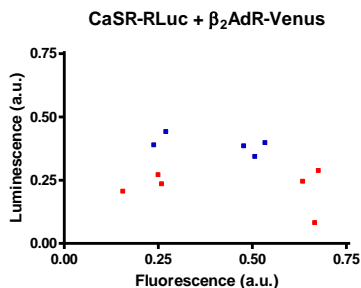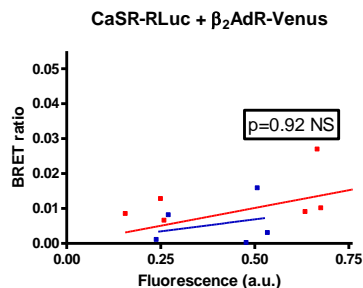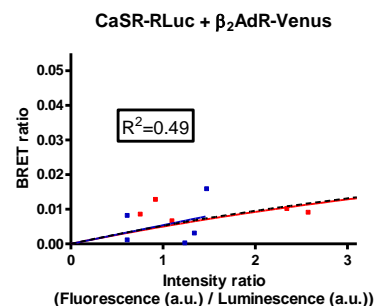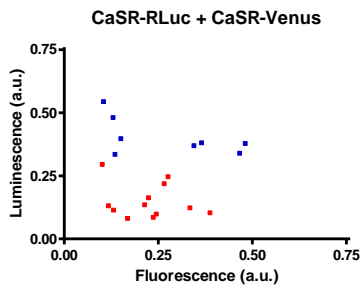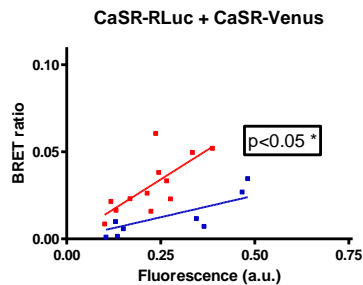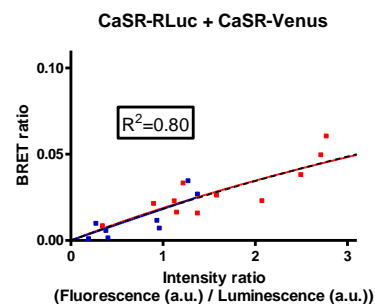

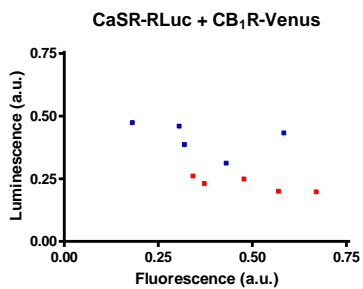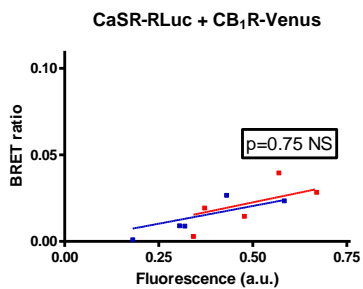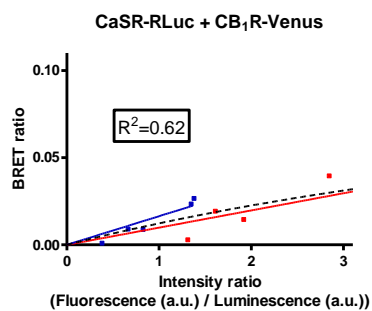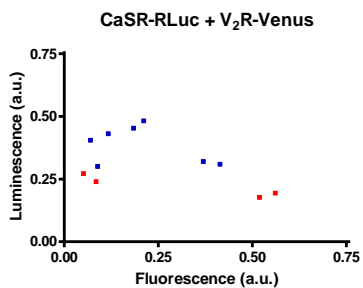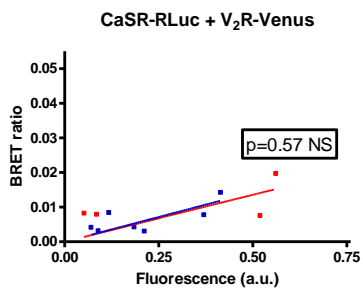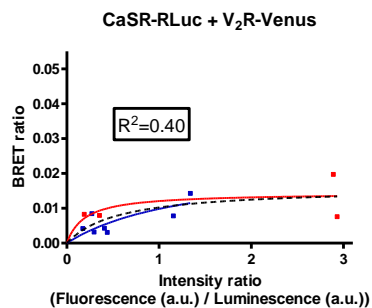

Supplement: Figure S2 — Fluorescence-Luminescence, Type I and Type II plots of GPCR dimerization experiments: HEK293 cells were transfected with various amounts of different donor and acceptor coding plasmids. Measured points were sorted into low/high luminescence groups based on the total measured luminescence (red: low luminescence, blue: high luminescence). Fluorescence-Luminescence (left), Fluorescence-BRET ratio (middle) and Intensity ratio-BRET ratio (right) plots were created for different donor-acceptor pairs. Summary of this plot can be found in Figure 4B and 4C. (PDF) [file pone.0109503.s002.pdf]
