# Supplementary material for: Correction: Improved Methodical Approach for Quantitative BRET Analysis of G Protein Coupled Receptor Dimerization
Source: PLoS One. 2016 May 31;11(5):e0156824. doi: 10.1371/journal.pone.0156824 (PMC4887035; doi:10.1371/journal.pone.0156824)
Supplement: S1 Table — (DOCX) [file pone.0156824.s001.docx]

S1 Table. Calculated p values for the difference between the slopes for high/low luminescence points

| **Acceptor** | **V_2_R-RLuc** | **CaSR-RLuc** |
| --- | --- | --- |
| **AT_1_R-mVenus** | 0.09 | 0.95 |
| **AT_2_R-mVenus** | 0.88 | 0.95 |
| **β_2_AdR-mVenus** | 0.51 | 0.95 |
| **CaSR-mVenus** | 0.88 | 1.6e-09 |
| **CB_1_R-mVenus** | 0.32 | 0.95 |
| **V_2_R-mVenus** | <2e-16 | 0.95 |

Linear model was fitted by forcing the regression line through the origin (lm(BRETratio~0+Acceptor:Fluorescence+Acceptor:Fluorescence:(Luminescence<threshold), using the statistical programming language R). Benjamini-Hochberg correction was performed on the p values for multiple hypothesis testing.
