# Supplementary figures and images for: Correction: Improved Methodical Approach for Quantitative BRET Analysis of G Protein Coupled Receptor Dimerization
Source: PLoS One. 2016 May 31;11(5):e0156824. doi: 10.1371/journal.pone.0156824 (PMC4887035; doi:10.1371/journal.pone.0156824)

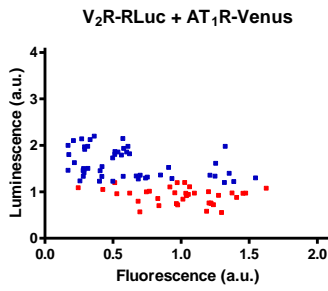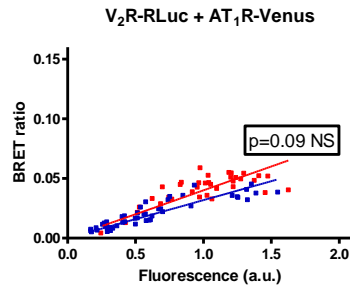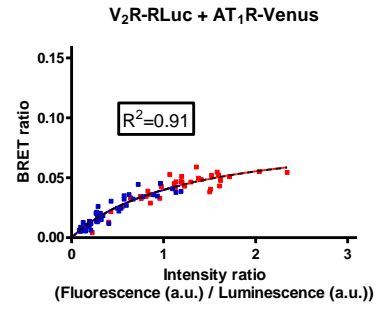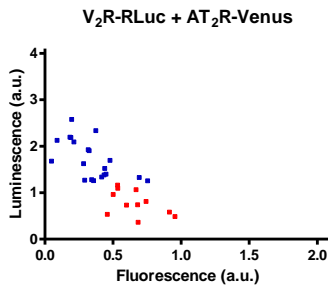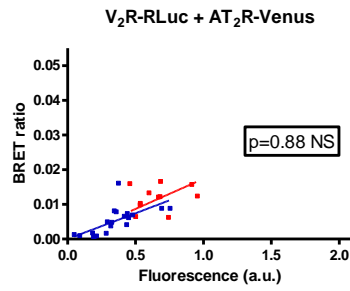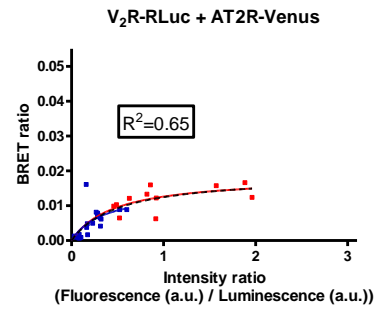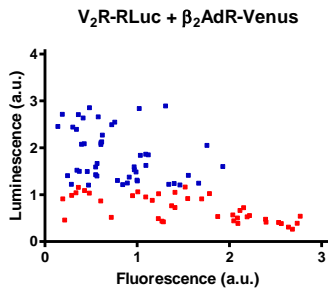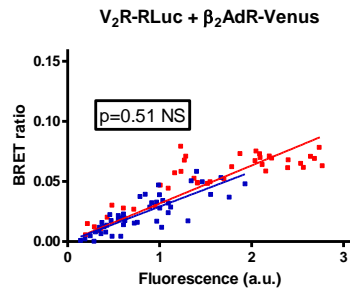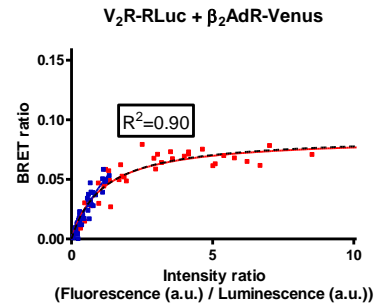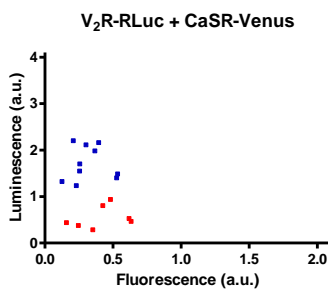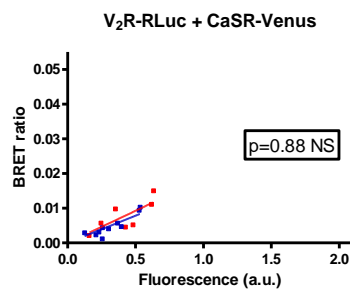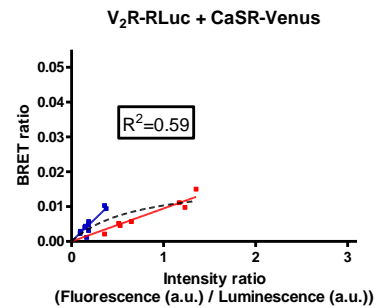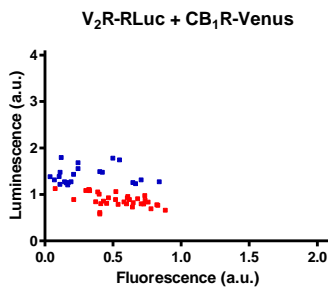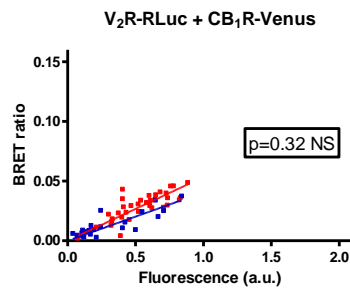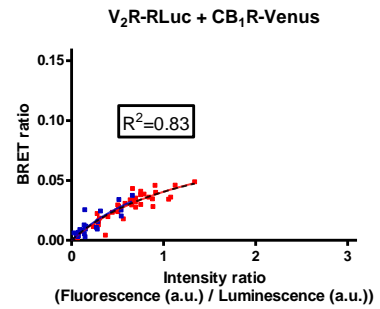

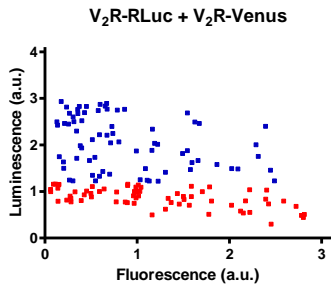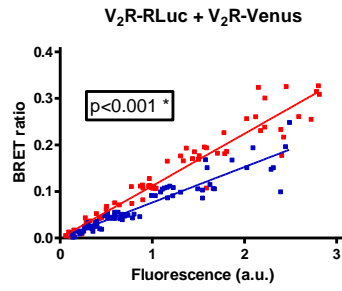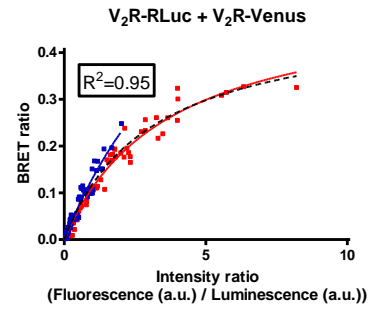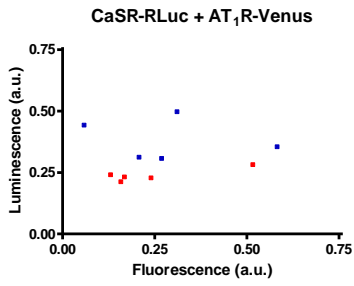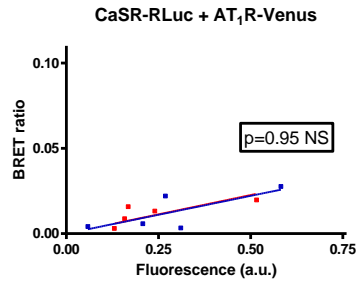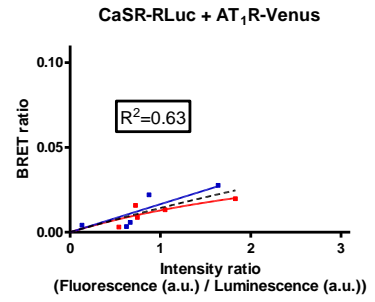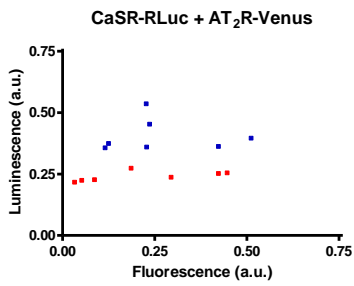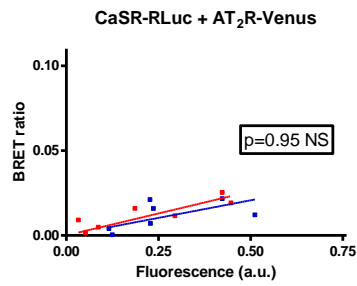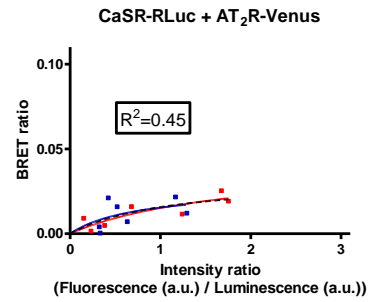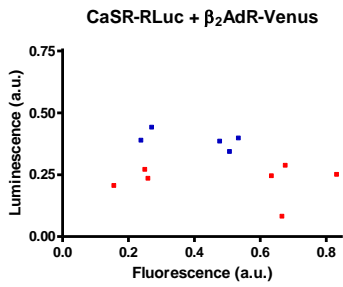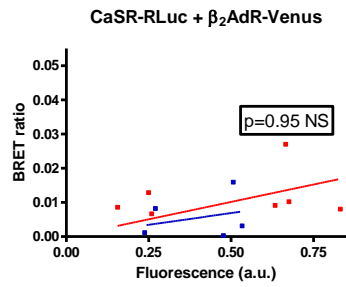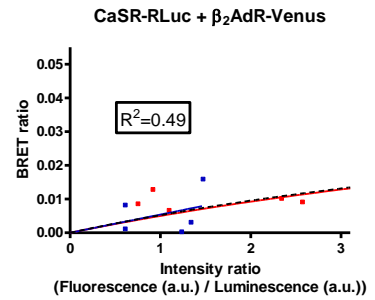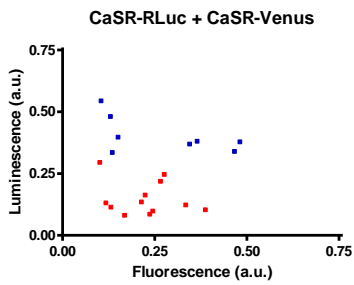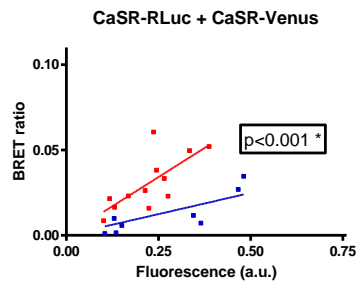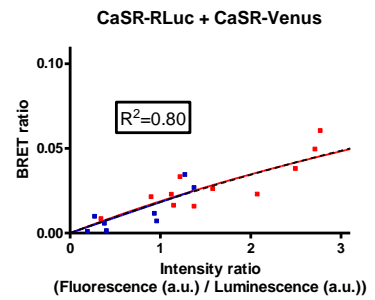

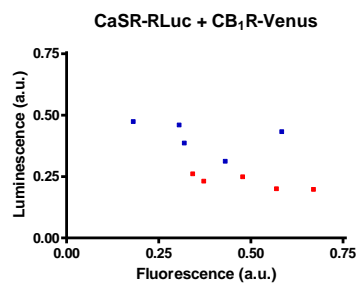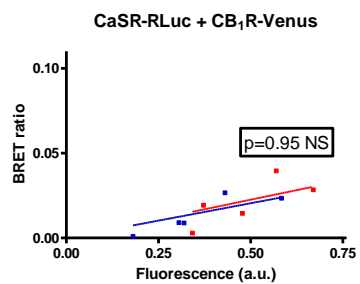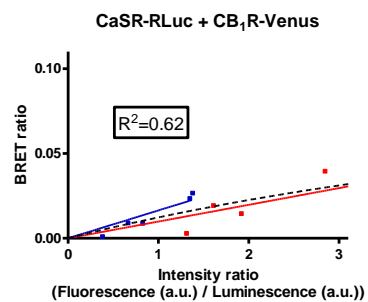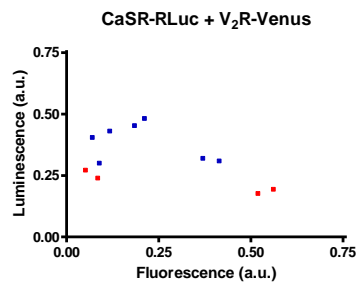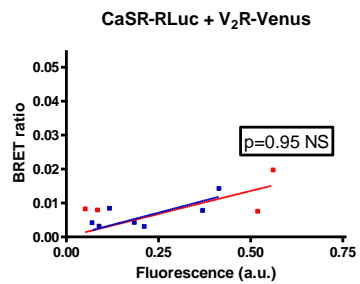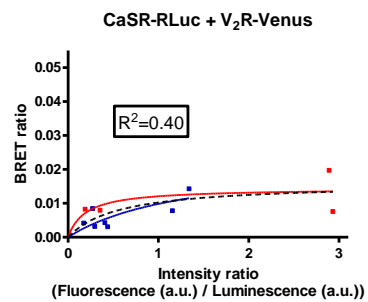

Supplement: S2 Fig — Measured points were sorted into low/high luminescence groups based on the total measured luminescence (red: low luminescence, blue: high luminescence). Fluorescence-Luminescence (left), Fluorescence-BRET ratio (middle) and Intensity ratio-BRET ratio (right) plots were created for different donor-acceptor pairs. Summary of this plot can be found in Fig 4B and 4C. (PDF) [file pone.0156824.s002.pdf]

### V<sub>2</sub>R-RLuc

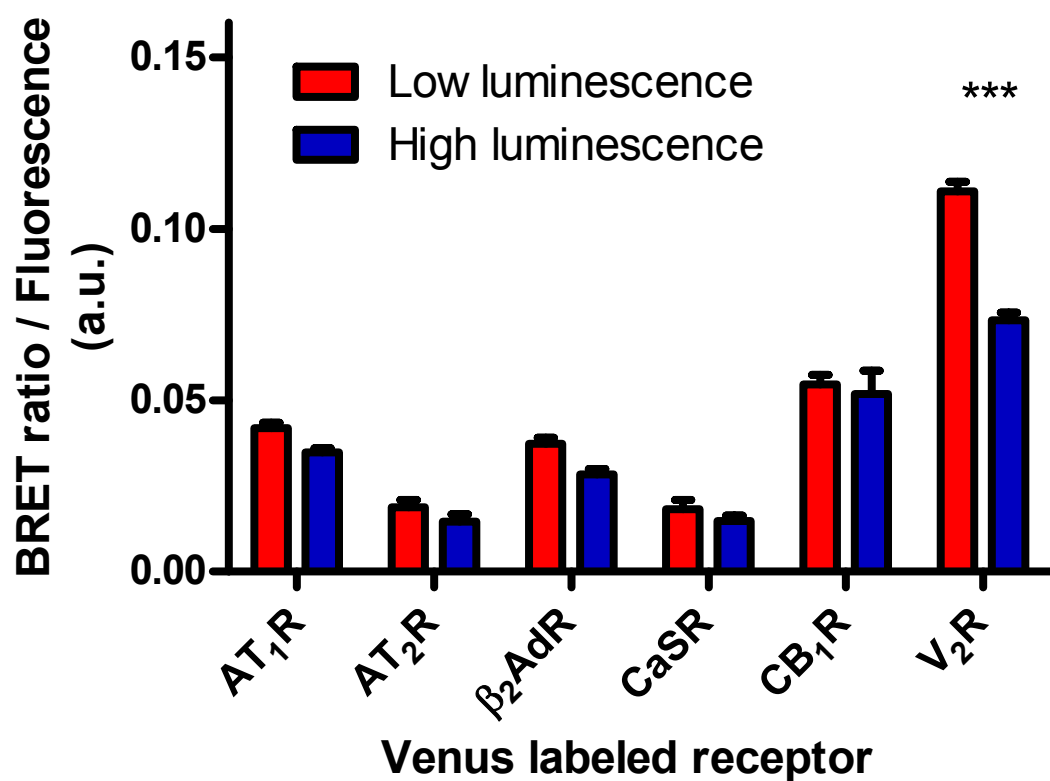

### CaSR-RLuc

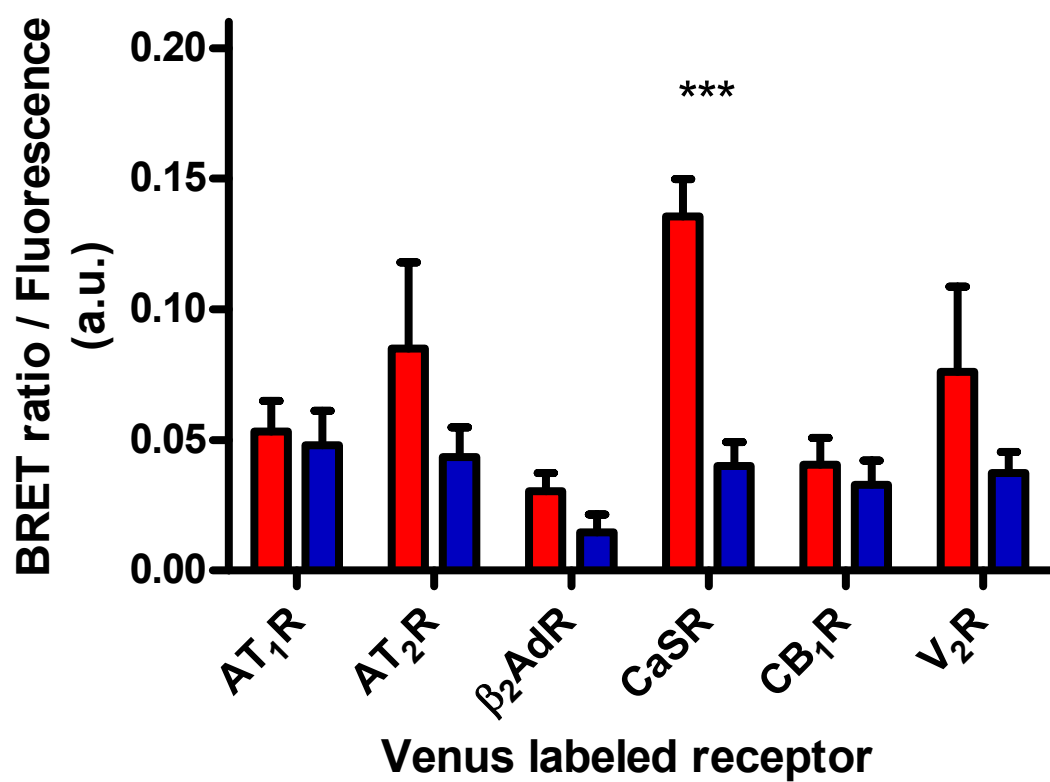

Supplement: S3 Fig — For each data point BRET ratio / Fluorescence ratio was calculated. Two-way ANOVA was performed (acceptor receptor and high/low luminescence as the two factors) with Bonferroni post hoc test on these data to evaluate the effect of high/low luminescence of BRET ratio / Fluorescence. Data are plotted as mean +/- S.E.M. ***: p<0.001 (PDF) [file pone.0156824.s003.pdf]
